# An Internet-Based Patient-Provider Communication System: Randomized Controlled Trial

**DOI:** 10.2196/jmir.7.4.e47

**Published:** 2005-08-05

**Authors:** Chen-Tan Lin, Loretta Wittevrongel, Laurie Moore, Brenda L Beaty, Stephen E Ross

**Affiliations:** ^2^Colorado Health Outcomes ProgramUniversity of Colorado at Denver and Health Sciences CenterDenverCOUSA; ^1^Division of General Internal MedicineUniversity of Colorado at Denver and Health Sciences CenterDenverCOUSA

**Keywords:** Ambulatory care, Internet, communication, patient satisfaction, randomized controlled trial

## Abstract

**Background:**

Internet-based interactive websites for patient communication (patient portals) may improve communication between patients and their clinics and physicians.

**Objective:**

The aim of the study was to assess the impact of a patient portal on patients’ satisfaction with access to their clinic and clinical care. Another aim was to analyze the content and volume of email messages and telephone calls from patients to their clinic.

**Methods:**

This was a randomized controlled trial with 606 patients from an academic internal medicine practice. The intervention “portal” group used a patient portal to send secure messages directly to their physicians and to request appointments, prescription refills, and referrals. The control group received usual care. We assessed patient satisfaction at the end of the 6-month trial period and compared the content of telephone and portal communications.

**Results:**

Portal group patients reported improved communication with the clinic (portal: 77/174 [44%] “a little better” or “a lot better;” control: 18/146 [12%]; χ^2^ = 38.8, *df* = 1, *P* < .001) and higher satisfaction with overall care (portal: 103/174 [59%] “very good” or “excellent;” control: 78/162 [48%]; χ^2^ = 4.1, *df* = 1, *P* = .04). Portal group patients also reported higher satisfaction with each of the portal’s services. Physicians received 1 portal message per day for every 250 portal patients. Total telephone call volume was not affected. Patients were more likely to send informational and psychosocial messages by portal than by phone. Of all surveyed patients, 162/341 (48%) were willing to pay for online correspondence with their physician. Of those willing to pay, the median amount cited was US $2 per message.

**Conclusions:**

Portal group patients demonstrated increased satisfaction with communication and overall care. Patients in the portal group particularly valued the portal’s convenience, reduced communication barriers, and direct physician responses. More online messages from patients contained informational and psychosocial content compared to telephone calls, which may enhance the patient-physician relationship.

## Introduction

The Institute of Medicine report “Crossing the Quality Chasm” [1] cites “the free flow of information” and “the patient as the source of control” as key features of patient-centered care. Information technology can play a vital role in meeting patient needs. Internet applications may be increasingly useful now that 73% of US adults have access to the Internet [2]. Previous studies have reported that 90% of patients with Internet access would like to communicate via email with their physician and that 56% indicate that it would influence their choice of doctor [3,4].

To meet this growing demand, some organizations have implemented Internet-based websites for communication between patients and their clinic and physician (patient portals) [4-10]. However, physicians have been hesitant about communicating online with patients, citing the potential for increased workload, increased medical liability, and risks to patient privacy [11-14].

Previous studies of Internet-based patient-provider communication include a randomized controlled trial [5] and several observational studies [4,6,11,12]. These studies established that online messaging between patients and physicians was an important medium of communication that was well accepted by patients yet used rarely by physicians. Although patient satisfaction with online messaging has been described, direct comparisons between online messaging and telephone call volume and content are lacking.

To better understand these issues, we conducted a randomized controlled trial while implementing a patient portal in an academic general internal medicine practice. This portal allowed patients to request appointments, prescription refills, and specialist referrals. It also allowed them to send secure electronic messages to their physician. We hypothesized that this portal would improve patient satisfaction with clinic operations. We were also interested in how the portal’s messaging system was used and how the volume and content of that use differed from telephone calls. Finally, we assessed the utility of the patient portal by asking patients to place a monetary value on its services.

## Methods

### Setting

The study was conducted at an academic ambulatory internal medicine practice affiliated with the University of Colorado Hospital in Denver, CO. Fourteen physicians and staff in the practice were already using an electronic medical record (EMR) (Care Innovation, version 8.0, 3M Health Information Systems, Salt Lake City, UT), which included an electronic messaging system to document patients’ incoming telephone calls. All 14 physicians participated in the study, answering messages from portal patients as well as controls. Clinic staff typed the content of incoming calls into the EMR. Staff nurses retrieved these messages electronically, called the patient, and documented their conversation or consulted the physician electronically prior to calling the patient. At baseline, physicians also communicated occasionally with patients using standard electronic mail (Outlook 2000, Microsoft Corporation, Redmond, WA). The volume of these email messages was small.

The study was approved by the Colorado Multiple Institutional Review Board.

### Recruitment and Randomization

Patients were enrolled from August 2002 through February 2003. The study period began at the conclusion of enrollment and lasted 6 months from March 1, 2003 through August 31, 2003. Eligible patients were at least 18 years old, English speaking, and had experience using an Internet browser. Patients were recruited via descriptive brochures, a poster, and a research assistant in the practice waiting room and via additional brochures in the examination rooms. Two broadcast emails were sent to 6000 employees of the University of Colorado Health Science Center, many of whom were eligible patients. An article about the study was also distributed to 2000 employees in the hospital’s newsletter. Patients were enrolled into the study after completing an online informed consent and initial survey.

Patients were consecutively assigned to intervention (access to the portal) or control (usual telephone care) groups by a research assistant according to a predetermined randomization scheme developed using SAS (SAS, version 8.2, SAS Institute Inc, Cary, NC), with equal numbers of portal and control participants in blocks of 10. Since patients in the portal group could send messages to physicians through the portal, physicians and clinic staff could not be blinded to the enrollment status of patients.

### Portal and Control Groups

**Figure 1:**
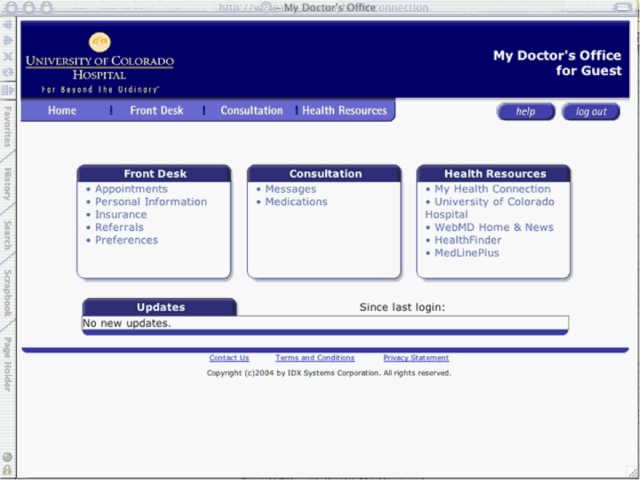
Screenshot of My Doctor’s Office portal

Portal group patients were instructed to register a username and password for the patient portal that the University of Colorado Hospital named “My Doctor’s Office” (Patient Online, version 6.0, IDX Systems Corporation, Burlington, VT). See Figure 1 and Multimedia Appendix 1 for screenshots of the portal website. They could then send requests for appointments, prescription refills, and referrals to the clinic staff and send clinical messages to their physician. Portal group patients were warned in advance not to send urgent messages using the portal. A clinic staff member copied incoming portal messages to the existing telephone messaging system three times daily (excluding weekends or holidays). Clinical messages were sent directly to the physician, who could send an electronic response to the patient or forward the message with instructions to clinic nurses. For patients in the control group, access to the portal was delayed until end of study. Instead, the control group received access to a website providing general health advice. Both portal and control patients could contact the clinic by telephone at their discretion or for urgent messages. The incoming telephone call triage system (for both portal and control patients) via the EMR was unchanged.

Broadcast emails were sent monthly during the study to patients in both groups. When these messages were returned due to an invalid email address, the research assistant attempted to retrieve correct contact information. Patients who could not be reached were disenrolled from the study. Also, portal group patients who had not registered their username and password were encouraged on five occasions via email to register.

### Outcome Measures

#### Patient Satisfaction

The primary outcome measures were patient satisfaction with the following: communication, overall care by the clinic, administrative requests (appointments, prescriptions, referrals), and clinical messaging (by portal and by telephone) with their physician. Patients completed surveys before and after the 6-month trial period (Multimedia Appendices 2-4). The initial survey assessed demographics and baseline satisfaction with clinic services. The final survey assessed satisfaction with clinic services for all participants and the perceived utility of the patient portal in the portal group. Satisfaction was assessed on a 5-point scale (1 = poor, 2 = fair, 3 = good, 4 = very good, 5 = excellent), with an option for patients to indicate that they “never did this.” Surveys were adapted by the investigators from prior instruments [4,6], pilot tested in a subset of nonstudy patients, and administered online. To maximize response rates, patients were contacted weekly by email for 4 weeks at the end of the 6-month trial period.

#### Qualitative Content Analysis of Administrative Requests and Clinical Messages

The patient portal tallied administrative requests and clinical messages. In all, 148/174 (85%) of portal patients and 142/166 (86%) of controls gave consent for investigators to review their medical record for the content of the clinical messages received via portal and telephone. The portal tracking system counted each incoming message separately, even if it was part of one clinical message exchange between patient and physician. During content review we only counted completed clinical message exchanges. We excluded clinical messages encompassing routine prescription refills, appointment requests, and referral requests from the content analysis.

We compared and categorized the content of clinical messages sent by patients in the portal and control groups. We adopted categories from a previous study [15] (request test information, request test action, request medication information, request medication action, miscellaneous) and added the following categories: urgent message, biomedical concern, psychosocial concern (eg, depression, anxiety, insomnia), FYI (for your information), home monitoring, and prevention. We identified message responses in which the physician elaborated on the advice given by the triage nurse and those in which the physician responded directly to the patient by a telephone call or portal messaging. Two of the investigators (CTL, LW) independently categorized message content according to this schema and met to resolve any interrater disparities.

#### Value to Patients

We asked patients whether they would pay for electronic correspondence with their doctor and, if so, to indicate a dollar value for this correspondence, per completed transaction. We calculated the median and mean dollar amounts provided by the patients who responded that they would pay for this correspondence.

### Statistical Methods

SAS was used for all statistical calculations. All tests of statistical significance were 2-tailed, with alpha = .05. With an anticipated sample size of approximately 300 participants per group and a 50% survey response rate, the study was designed to have 80% power to detect a difference of 15% in the proportions of the portal group and control group who rated overall communications with the clinic as “a lot better.”

Data were examined to determine frequencies and normality of responses. Baseline characteristics and outcomes for both groups were assessed with Student *t*-tests or nonparametric Wilcoxon rank sum tests for count variables and chi-square tests for categorical variables or Fisher exact tests when appropriate. The nonparametric Kendall tau rank correlation was used to assess the correlation between categorical variables.

## Results

The complete study data are included in Multimedia Appendix 5.

### Participants

Demographic characteristics of the portal and control groups were comparable at the beginning of the study. Over 70% of the participants in both groups were college graduates and had a household income of at least US $45000 (Table 1). Proportionately, 96/305 (31%) of portal patients and 111/301 (37%) of controls registered using “uch.edu” or “uchsc.edu” email addresses and could be identified as employees of the University of Colorado Hospital or Health Sciences Center (χ^2^ = 2.0, *df* = 1, *P* = .16). This was reflective of the general patient population at the study clinic.

**Table 1:** Baseline demographics

	**Portal Group** **n = 305** **Mean (SD) or No. (%)**	**Control Group** **n = 301** **Mean (SD) or No. (%)**	***t* or χ^2^**	***P* value**
Mean age, years	52 (13)	50 (12)	-1.6[Table-fn table1fn1]	.12
Women	175 (57%)	176 (59%)	0.1[Table-fn table1fn2]	.75
College graduate and above	215 (75%)	224 (78%)	0.5[Table-fn table1fn2]	.46
Income ≥ US $45000/year	215 (76%)	221 (76%)	0.004[Table-fn table1fn2]	.95

Degrees of freedom = 585

Degrees of freedom = 1

**Figure 2:**
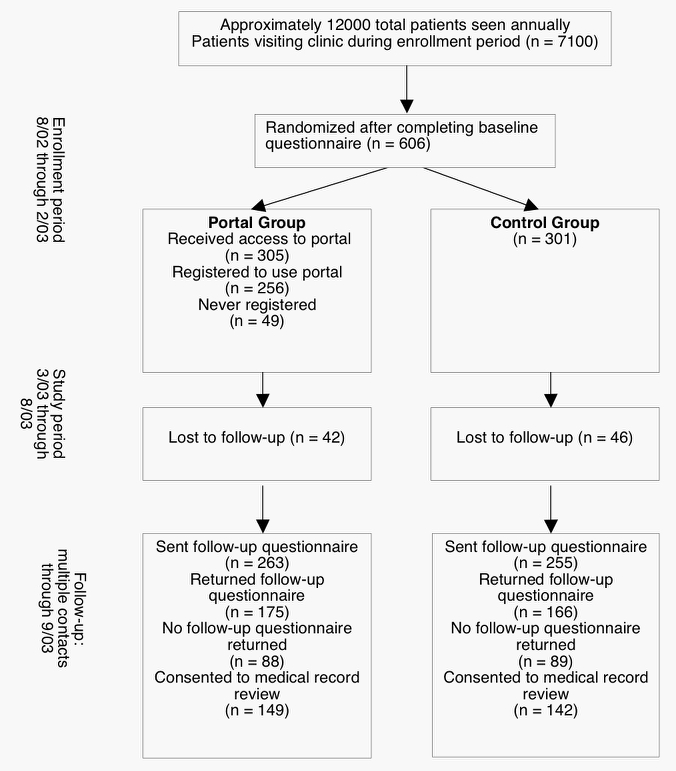
Flow of participants throughout study period

In all, 606 patients completed a baseline questionnaire. Of the 305 patients who were allocated to the portal group, 256 (84%) obtained a user account for the patient portal, and 95 (31%) used the portal during the trial period (Figure 2).

A similar proportion of participants in the portal and control groups were lost to follow-up: 42 (14%) in the portal group and 46 (15%) in the control group. Of those who were sent the final survey, 175 (67%) of portal patients and 166 (65%) of controls responded. We compared overall satisfaction with care on the initial survey between participants who completed the study and those who did not (defined as those lost to follow-up plus those who never completed the final survey). Those who did not complete the study were less satisfied on the initial survey compared to those who did complete the study (completed study: 137/341 [40%] reported last interaction with clinic as “very good” or “excellent;” did not complete study: 84/265 [32%]; χ^2^ = 7.3, *df* = 1, *P* = .007). However, of those who completed the study, there was no significant difference in initial satisfaction with last clinic interaction between the portal group and controls (portal group: 106/305 [35%] reported last interaction with clinic as “very good” or “excellent;” control group: 115/301 [38%]; χ^2^ = 0.3, *df* = 1, *P* = .57).

### Outcomes

#### Patient Satisfaction

At the end of the 6-month study, portal group patients were more likely to indicate that communication with the clinic had improved, and they were more likely to rate clinic services as “very good” or “excellent” compared to controls. Portal group patients were also more satisfied than controls with overall clinic services, and, for those portal group patients who used the administrative services, they were more satisfied with each of the services (appointments, refills, and referrals) and with clinical messaging (Table 2).

In subgroup analysis, portal group patients who never used the portal (portal group nonusers) were similar to controls. The only “overall service” in which portal group nonusers differed from controls was satisfaction with “current communication with the clinic compared with the beginning of the study.” The only “specific service” in which portal group nonusers differed from controls was in the subgroup of patients who scheduled appointments.

**Table 2:** Patient satisfaction at the end of 6-month study period

**Question**		**Control** **n = 166** **No. (%)**	**Portal Group Overall** **n = 175** **No. (%)**	**Portal Group Nonuser** **n = 98** **No. (%)**	**Portal Group Overall vs Control**		**Portal Group Nonuser vs Control**	
					**χ^2[Table-fn table2fn1]^**	***P* value**	**χ^2[Table-fn table2fn1]^**	***P* value**
**Compared with the beginning of the study, would you say that your communication with the clinic is…** (a little better/a lot better)		18 (11%)	77 (44%)	29 (30%)	38.8	< .001	11.2	< .001
**Overall, how would you rate the services you receive from the clinic?** (very good/excellent)		78 (48%)	103 (59%)	49 (50%)	4.1	.04	0.1	.77
**Based on your experiences using the phone or the portal to contact the clinic, please rate the services below:**						
	Communicating nonurgent messages to doctor and/or nurse (very good/excellent)	n = 137 43 (31%)	n = 141 77 (55%)	n = 76 32 (42%)	15.3	< .001	2.5	.12
	Refilling prescription (very good/excellent)	n = 118 52 (44%)	n = 95 60 (63%)	n = 45 24 (53%)	7.7	.006	1.1	.29
	Requesting referrals (very good/excellent)	n = 106 44 (42%)	n = 80 50 (63%)	n = 43 24 (56%)	8.0	.005	2.5	.11
	Scheduling appointments (very good/excellent)	n = 154 47 (31%)	n = 131 71 (54%)	n = 70 31 (44%)	16.4	< .001	4.0	.045

Degrees of freedom = 1

Patient satisfaction with the portal was high: of the 114/175 (65%) who reported using the portal, 80 (70%) indicated that they were “satisfied” or “very satisfied” with portal services, 92 (81%) indicated that it saved them a telephone call to the clinic, and 37 (33%) indicated that it saved them a visit to the clinic during the 6-month trial.

Of the entire portal group, 132/175 (75%) indicated they were “likely” or “very likely” to use the portal in the future, and 149 (85%) said they would prefer to use the portal over the telephone for nonurgent messages.

To determine whether frequent users of the portal were more satisfied with clinic services, we evaluated the association between the number of times patients logged on to the portal and their responses to questions about satisfaction. There were weak positive correlations between frequency of portal use and the following: satisfaction with portal services (*r* = 0.18, *P* = .02), improved communication with the clinic since baseline (*r* = 0.19, *P* = .01), and, for those who used it (n = 99), satisfaction with physician messaging (*r* = 0.17, *P* = .03).

##### Qualitative Content Analysis of Administrative Requests and Clinical Messaging

**Figure 3:**
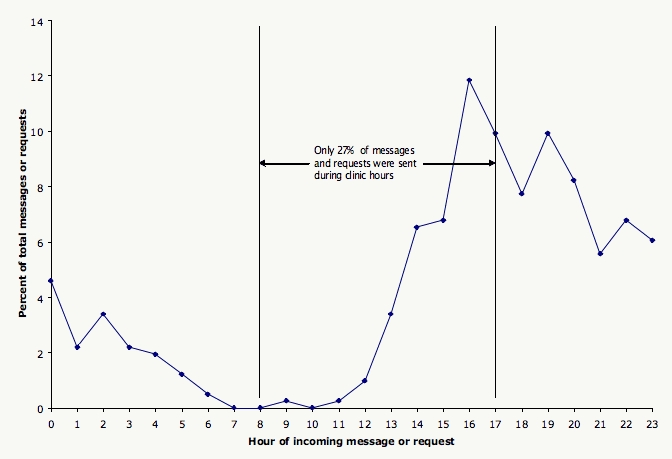
Incoming patient portal administrative requests and clinical messages by hour of the day (n = 414)

The 95 patients who actually used the portal sent a total of 175 administrative requests (88 appointments, 72 prescription refills, and 15 referrals) and 239 clinical messages. This translated to physicians receiving, on average, 1 clinical message per day for every 250 portal group patients enrolled. Monthly volumes were stable over the course of the study. Of these requests and messages, 27% were sent during clinic hours, and 73% were sent during nonclinic hours (Figure 3). Moreover, 52% were sent from 5 pm to midnight, 12% were sent from midnight to 8 am, and 9% were sent on weekends or holidays. Although not explicitly measured, clinic staff spent about 8 hours daily answering telephones and about 5 minutes daily triaging and responding to portal requests and messages.

We compared the clinical messages of patients in both study groups who consented to a review of their medical record. Portal patients called 110 times and sent 76 portal messages. Control patients called 126 times. The median number of telephone calls per patient and total messages (telephone plus portal) per patient was 0 for both the portal and control groups. There was no significant difference in the number of telephone calls (*U* = 1.1, *P* = .26) or total number of contacts (*U* = -1.1, *P* = .29) between the portal and control groups. The mean number of telephone messages per patient was 0.36 (SD = 1.25) in the portal group and 0.42 (SD = 1.06) in the control group, and the mean number of total (telephone plus portal) messages per patient was 0.61 (SD = 1.79) in the portal group and 0.42 (SD = 1.06) in the control group (this is the same as the number of telephone calls since this is the only way this group contacted the clinic). In aggregate, clinical messages from portal patients and controls were similar in content (data not shown).

Within the portal group, we also compared the content of clinical messages sent by portal and by telephone (Table 3). Patients sent more FYI (for your information) and psychosocial messages via the portal. Only 2 portal messages were deemed urgent, and the receiving physicians did not consider these problematic.

**Table 3:** Content of clinical messages from portal group (comparing phone and portal messages)

**Type of Message**	**Description of Message**	**Clinical Phone Messages** **n = 110[Table-fn table3fn1]**	**Clinical Portal Messages** **n = 76[Table-fn table3fn1]**	**χ^2[Table-fn table3fn2]^**	***P* value**	**Portal Clinical Message Examples**
Urgent message	Patient expects same-day return call and describes acute symptoms or symptoms of infection, or patient calls twice in one day for the same reason	37 (34%)	2 (3%)	26.1	< .001	“I woke up 3:30 am with pain in left side, assumed it was a kidney stone. Took one Dilaudid 2 mg.... If it persists, more than 24 hours I will seek medical care unless you advise sooner.”
Biomedical concern	Patient describes body symptoms such as fever, nausea, pain, headache, sore throat, dizziness	57 (52%)	32 (42%)	1.7	.19	“Several years ago I strained the lower left quadrant of my back. Periodically, I strain it and Advil usually reduces the swelling/pain. Last week, I strained it again, and I was hoping you could prescribe something that could help. Thanks.”
Psychosocial concern	Patient describes symptoms such as stress, anxiety, depression	2 (2%)	7 (9%)	5.3	.02	“I saw you yesterday for a variety of issues but didn’t mention that I am dealing with some heavy depression. I did start Prozac again.... I am having difficulty eating, and am crying a lot. This may also relate to the intestinal issues I am having... (gosh, I’d like to be sleeping at this 3:00 am hour).”
Request test information	Patient requests the results of tests or asks a question about results	12 (11%)	13 (17%)	1.5	.22	“I looked at my test results and I have a few questions. They were supposed to run a Hep C, and LFTs. I assume my Hep B antibody came back positive due to the fact that I was immunized. Please let me know if I need to get more blood drawn.”
Request test action	Patient requests that a test be done (eg, blood test, urine culture, x-ray)	9 (8%)	5 (7%)	0.2	.68	“Anything else you think I should get done before I see you? Is it time to do a full blood work—hormones, cholesterol, etc?”
Medication question	Patient requests information about a medication	8 (7%)	1 (1%)	3.5	.08	“What’s your opinion of fluoxetine and/or bupropion as antidepressants? They are reported to have virtually no side effects relative to the other meds used for depression.”
Request medication action	Patient requests a change in medication dose or new medication	33 (30%)	16 (21%)	1.9	.17	“What I’ve been left with is a persistent cough...and very heavy sinus drainage, pretty much all day and all night.... I start rehearsals in a few weeks, and it’s going to be very difficult for me to work unless I can get rid of this cough and sinus drainage. Can you think of any treatment that might help?”
FYI (for your information)	Patient provides new ideas or information to the physician not otherwise categorized	2 (2%)	14 (18%)	15.8	< .001	“Well, I think I might have solved my hive problem...bed bugs. I was away for a few days and did not have any new hives.... Last night I found some bugs in my bed. I have a pretty bad outbreak of hives again....
Home monitoring	Patient reports personal health data (eg, blood sugar, blood pressure, weight, exercise)	3 (3%)	4 (5%)	0.8	.45	“Here are some BP readings for the mornings of January. I started taking my diuretic on the third.”
Prevention	Patient requests screening tests or procedures such as colonoscopy or immunization	1 (1%)	4 (5%)	3.3	.16	“I do want to get a bone density test at some point but rib seems fine now and am not looking forward to any more tests.”
Miscellaneous	Patient makes a request regarding a medical excuse, insurance issue, document, etc.	13 (12%)	14 (18%)	1.6	.21	“I was denied for Preferred Life Insurance recently—something I have had for over ten years—because of my recent medical problems.”

Total percents exceed 100 as messages may contain topics in more than one category.

Degrees of freedom = 1

When physicians responded to clinical messages from the portal group, they were more likely to elaborate on the advice of the triage nurse when the message was received through the portal (physician input to portal message: 73/76 [96%]; physician input to phone message: 86/110 [78%]; χ^2^ = 11.6, *df* = 1, *P* < .001). They were also more likely to respond directly to the patient (physician direct response to portal message: 60/76 [79%]; physician direct response to phone message: 15/110 [14%]; χ^2^ = 79.7, *df* = 1, *P* < .001).

##### Value to Patients

In all, 162/341 (48%) of all survey respondents were willing to pay for electronic correspondence with their doctor. Of those willing to pay, the median amount reported per message was US $2, and the mean was $4.10.

## Discussion

In this randomized controlled trial, patients with access to an Internet-based patient portal were more satisfied with their communication with the clinic and their overall care. These patients were also more satisfied with clinical messaging with their physicians and the process of requesting appointments, prescription refills, and referrals. Patients were more likely to send FYI and psychosocial messages via the portal than by telephone. The volume of incoming messages was minimal: 1 message daily for every 250 patients offered online access. Portal and control group clinical message volumes were not significantly different.

Why were portal patients more satisfied than controls? First, the portal was convenient: 81% believed that the portal saved them a telephone call, and 33% believed it saved them a visit to the clinic. The portal allowed patients to send messages at all hours; indeed, 73% of incoming messages were sent during nonclinic hours. Second, the portal reduced barriers to communication—portal patients were more likely to send FYI and psychosocial messages. Patients may hesitate to “bother the doctor” with FYI messages by phone, but they feel more comfortable sending a portal message. Patients may prefer sending psychosocial messages by portal because it affords privacy and distance, avoiding the aggravation of being on hold and having to speak to an intermediary. One patient even suggested to the physician that the portal was a more comfortable medium for psychosocial discussion than in-person visits. Third, patients may have appreciated that portal messages were more likely to receive direct responses from the physician, whereas telephone calls tended to be mediated by a triage nurse. Finally, the portal was efficient, providing quick message responses that likely exceeded patient expectations. A substantial majority of messages were answered the same day, even though the portal states that responses may take up to 2 business days. This is consistent with other studies that demonstrated improved patient satisfaction with shorter response time [11] and with meeting or exceeding patient expectations [16,17].

It is clear that patients increasingly desire and are satisfied with online messaging. Physicians are much less enamored with electronic communication, driven by fears of overwhelming volume, inappropriate messaging, and inadequate security [5,10,18,19]. The increasing publicity of patients demanding such service, the lack of demonstrated adverse effects, and the possibility of insurers reimbursing physicians for online communication may narrow this satisfaction gap [8,18,20].

In our sample, the total number of incoming messages from portal patients (portal plus phone) was not significantly different from the total number of incoming messages from controls. This implies that patients replace phone calls with electronic messages. Although Katz et al [5] showed that total message volume increases with patient access to online messaging, others have shown a replacement of phone calls with online messages [4,18,19]. Although not specifically measured, both physicians and staff noted that responding to electronic messages took less time than responding to telephone messages, even after discounting the frustration of “telephone tag.” Others have corroborated this finding [18].

Portal patients called more times than they sent online messages. Why? Urgent calls were one third of the phone call volume. Subtracting urgent calls, portal patients were equally likely to call as they were to send an online message. Adopting a new communications medium may occur gradually, with patients not trusting the new system, forgetting how to access it, or not thinking to use it.

The clinical utility of incoming messages is beyond the scope of this study. It is not clear, for example, how FYI messages might have impacted care. At worst, one might imagine such messages “cluttering” the patient’s medical record. At best, it might “close the loop” when patients inform their physician of the success or failure of a treatment. Although we demonstrated improvement of patient satisfaction, we are unable to state whether quality of care was affected.

Portal group patients who never used the system were similar to controls in their satisfaction with clinic services, except that they were more satisfied with “overall communication.” These patients may have felt that simply having the portal available if they needed it was advantageous.

Some organizations are charging patients for portal clinical messaging. Since 53% of study participants would not pay for portal messages, this could shift portal messages to “free” telephone calls, reduce FYI and psychosocial messages, and affect satisfaction. Notably, some insurers are beginning to reimburse physicians for online communications, which may partially address this concern [8].

### Limitations

This study has several limitations. Control group patients who continued emailing their physician may have diluted the difference between groups. Our patients had relatively high incomes, educational status, and familiarity with the Internet, and one third were University of Colorado or Health Science Center employees, so these results may not be generalizable to an Internet-naive, less affluent sample. Because of the nature of the intervention (online messaging vs telephone calls), the physicians and staff could not be blinded to the process and may have paid more attention to online messages, influencing the results. The study was conducted for only 6 months; patient satisfaction could have increased (due to increased familiarity) or decreased (due to fading of initial enthusiasm) if the study was carried out over a longer period of time. We note that our final sample size (N = 95 actual users) was smaller than our desired size of 150. We reported that the portal group achieved higher satisfaction than controls for “overall care” (*P* = .04). A larger sample would have provided a more precise estimate of effect. Lastly, because of our recruitment method, we were unable to collect information from patients who were eligible for the study but declined to participate. Despite initial randomization of patients and comparable demographic characteristics in dropouts, those who dropped out of the portal and control groups may be different, biasing the results of the final survey.

### Summary and Future Directions

This randomized controlled study adds to the literature by describing possible underlying reasons for patient satisfaction with online communication: convenience, reduced communication barriers, and direct physician responses. Another novel finding was that more online messages from patients contain FYI and psychosocial content compared to telephone calls. These findings may explain why patient access to an Internet-based patient portal was associated with greater patient satisfaction with communication and overall care.

A patient portal that supports online communication is a strong foundation on which to promote “care based on continuous healing relationships” [1] and encourage collaborative care. Further research is needed to evaluate more advanced portal services and their impact on patient satisfaction, empowerment, and medical outcomes. An advanced patient portal might include shared documentation by physicians and patients, patient access to test results and other aspects of their medical record, and shared decision support to patients and physicians for chronic care improvement.

## Multimedia Appendix 1

Screenshots of My Doctor’s Office portal.

## Multimedia Appendix 2

Baseline patient satisfaction questionnaire.

## Multimedia Appendix 3

Final satisfaction questionnaire for subjects.

## Multimedia Appendix 4

Final satisfaction questionnaire for controls.

## Multimedia Appendix 5

Complete study data.
