# Supplementary figures and images for: An Internet-Based Patient-Provider Communication System: Randomized Controlled Trial
Source: J Med Internet Res. 2005 Aug 5;7(4):e47. doi: 10.2196/jmir.7.4.e47 (PMC1550679; doi:10.2196/jmir.7.4.e47)

## Slide 1
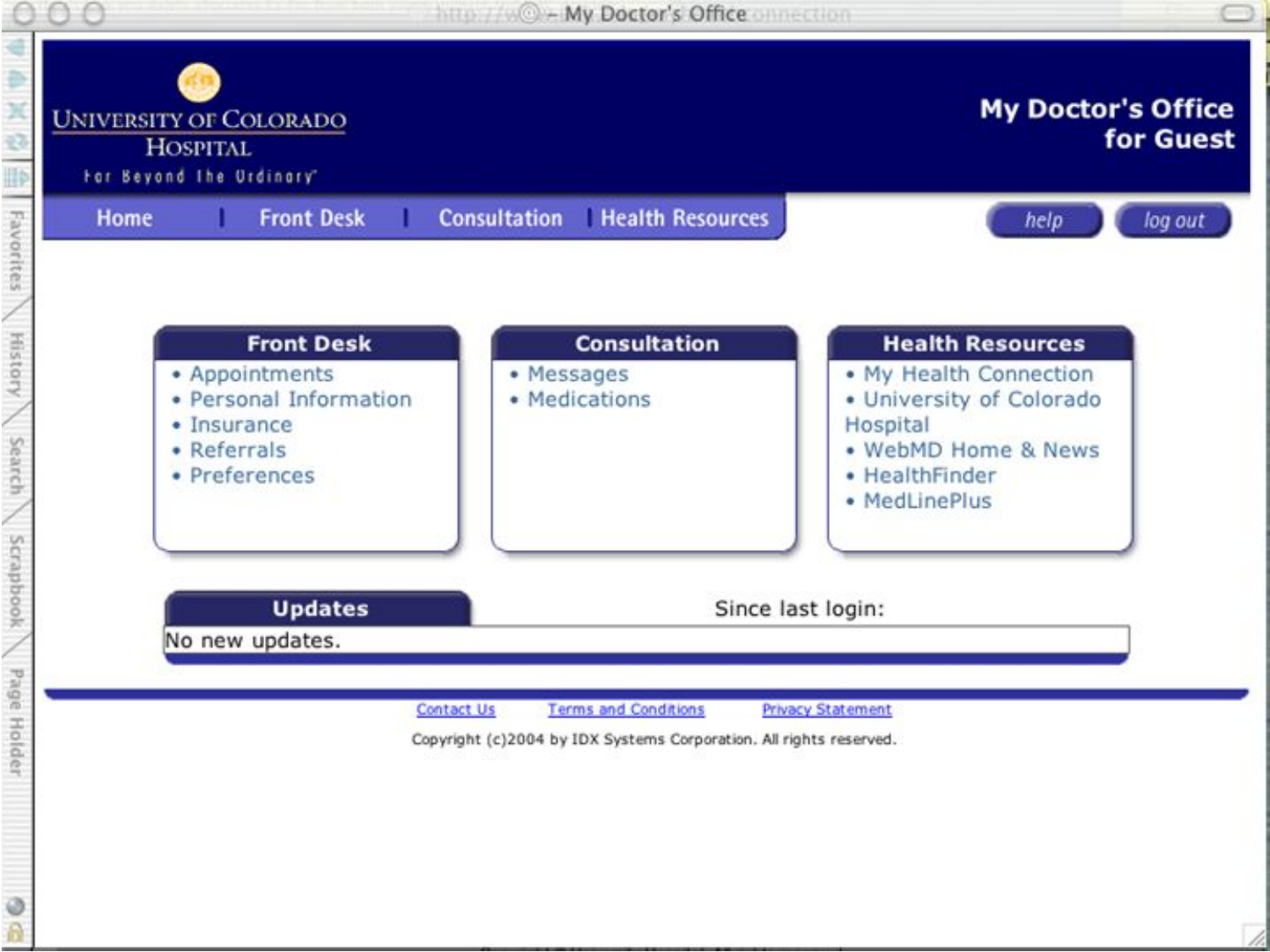

#

## Slide 2
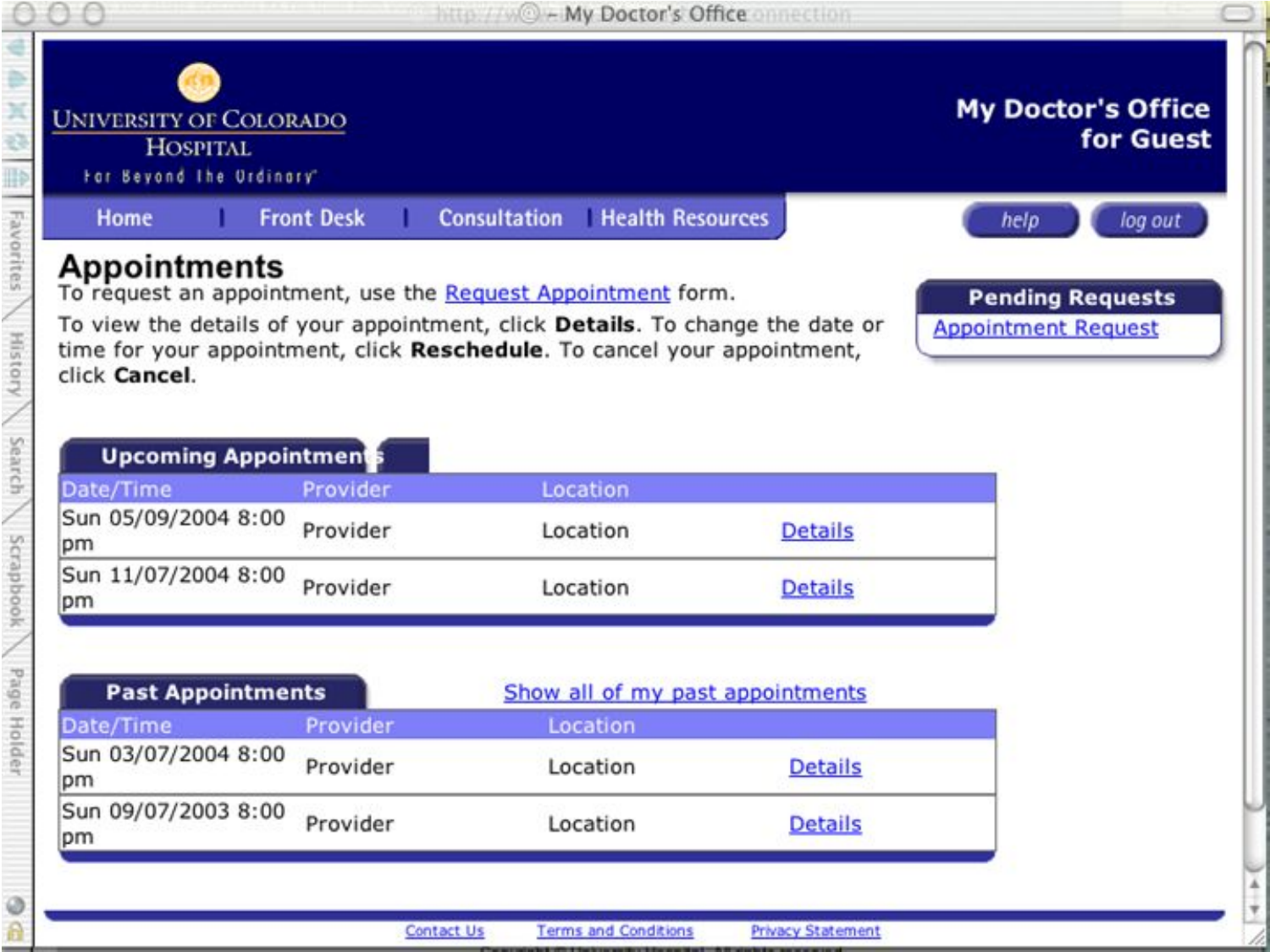

#

## Slide 3
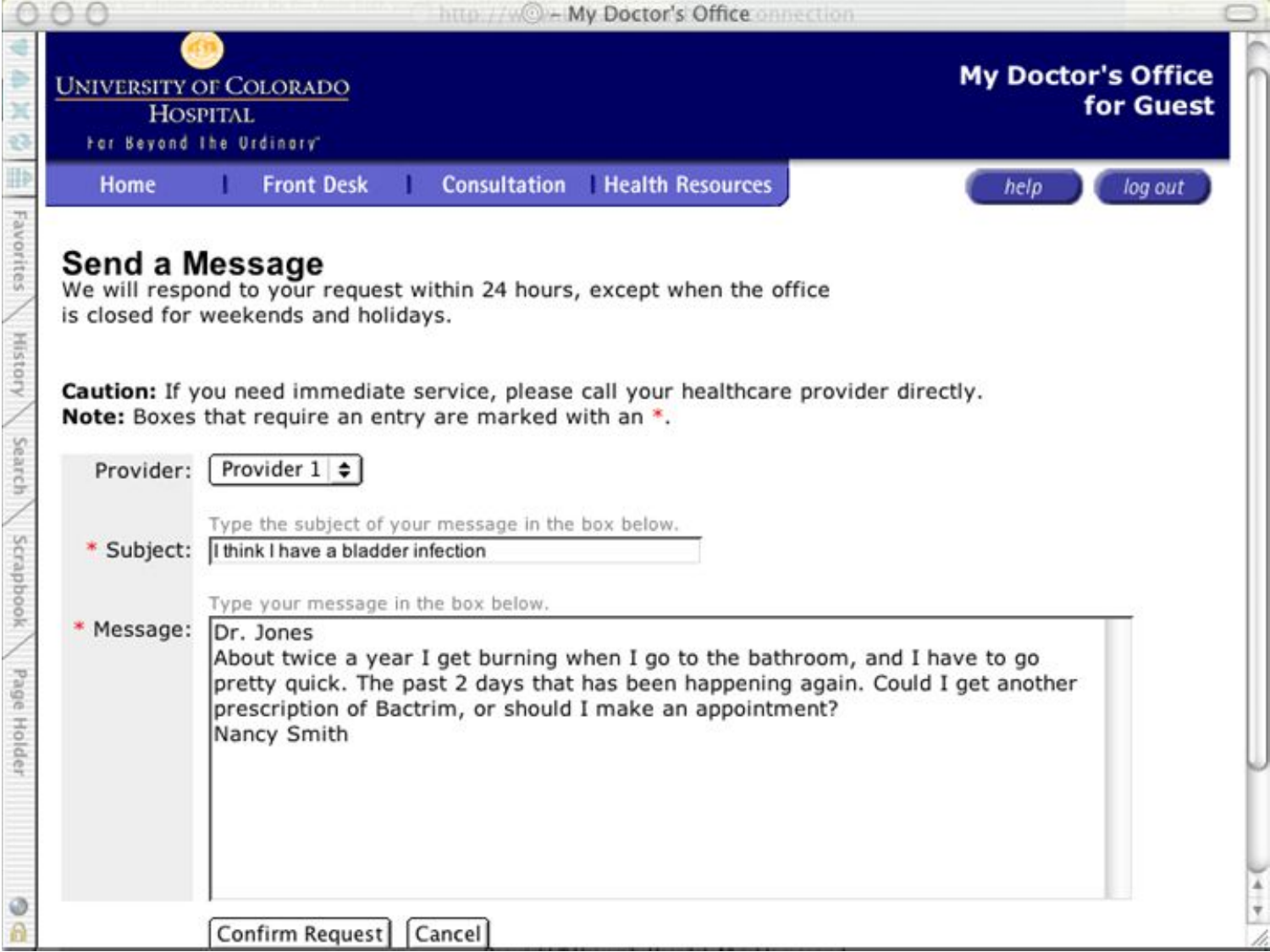

#

Supplement: Supplementary file 1 [file jmir_v7i4e47_app1.ppt]
