# Supplementary material for: An Internet-Based Patient-Provider Communication System: Randomized Controlled Trial
Source: J Med Internet Res. 2005 Aug 5;7(4):e47. doi: 10.2196/jmir.7.4.e47 (PMC1550679; doi:10.2196/jmir.7.4.e47)
Supplement: Supplementary file 2 [file jmir_v7i4e47_app2.pdf]

My Doctor's Office – BASELINE

The following questions relate to your interaction with the clinic. Point the arrow and click the box that represents the best answer.

1. In the past 6 months, how many times did you call the clinic?

❑ 0 ❑ 1 ❑ 2 ❑ 3 ❑ >3

2. In the past 6 months how many times did you email the clinic staff or your physician (not while in My Doctor's Office)?

❑ 0 ❑ 1 ❑ 2 ❑ 3 ❑ >3

3. In the past 6 months, how many times were you seen in the clinic by either a doctor or nurse?

❑ 0 ❑ 1 ❑ 2 ❑ 3 ❑ >3

4. In the past 6 months, what was your most recent interaction with the clinic, **excluding a doctor visit**?

❑ 1 Phone call
❑ 2 Electronic mail (e-mail)
❑ 3 Did not contact the clinic in the past 6 months
❑ 4 Other

If you did not contact the clinic in the past 6 months, please skip questions 6 and 7. Go to question 8 and continue from there. (Questions 6 and 7 do not apply to you)

5. What was your reason for contacting the clinic? *(check only one)*:

❑ 1 Schedule an appointment
❑ 2 Ask for a referral
❑ 3 Ask a question
❑ 4 Prescription refill
❑ 5 Other

6. Based on your response to question 5 above, what is your overall rating of your most recent clinic interaction?

❑ Poor	❑ Fair	❑ Good	❑ Very good	❑ Excellent
1	2	3	4	5

7. For which of the following services would you prefer to use a secure website such as My Doctor's Office rather than the telephone? *(check all that apply)*

❑ Communicate non-urgent messages for doctors and/or nurse
❑ Refill prescriptions
❑ Request referrals
❑ Schedule appointments
❑ Other

8. If you would prefer using a secure website such as My Doctor's Office, please tell us why. *(write in the space provided below)*

Please provide the following information on your background. All of this information will be kept completely confidential.

10. **What year were you born?** | 19 |

For questions 11 to 13, check only one answer:

11. **Are you male or female?**

❑ Male
❑ Female

12. What is the highest grade or year of school you completed?

❑ Grades 1-8
❑ Grades 9-11/Some high school
❑ High school graduate
❑ Some college/university
❑ College graduate
❑ Post-graduate education or degree

13. **What is your household income per year?**

❑ Less than $15,000 per year
❑ $15-29,000
❑ $30-44,000
❑ $45-59,000
❑ $60-74,000
❑ $75-90,000
❑ over $90,000

You have successfully completed this questionnaire.

Thank you for your participation.

Your comments are welcome. Please write in the box below.
