# Supplementary material for: An Internet-Based Patient-Provider Communication System: Randomized Controlled Trial
Source: J Med Internet Res. 2005 Aug 5;7(4):e47. doi: 10.2196/jmir.7.4.e47 (PMC1550679; doi:10.2196/jmir.7.4.e47)
Supplement: Supplementary file 3 [file jmir_v7i4e47_app3.pdf]

My Doctor's Office (MDO) Electronic Survey #2.[illegible] Follow-up Survey for Subjects

First Name ________ Last Name ________

Email ________

The following questions relate to your interaction with the clinic. Point the arrow and click the box that represents the best answer.

1. In the past 6 months, how many times did you call the clinic?

0 ☐ 1 ☐ 2 ☐ 3 ☐ more than 3 ☐

2. In the past 6 months how many times did you send email (not using My Doctors Office online service) to the clinic staff or your physician?

0 ☐ 1 ☐ 2 ☐ 3 ☐ more than 3 ☐

3. In the past 6 months, how many times were you seen in the clinic by either a doctor or nurse?

0 ☐ 1 ☐ 2 ☐ 3 ☐ more than 3 ☐

4. In the past 6 months, what was your most recent interaction with the clinic, excluding a doctor visit? *(check only one)*

☐ Phone call
☐ Electronic-mail (e-mail) not using My Doctor's Office (MDO) online service
☐ Messaging using MDO online services
☐ Other ____________________
☐ Did not contact the clinic in the past 6 months

5. **What was your main reason for contacting the clinic?** *(check only one)*

☐ Schedule an appointment
☐ Ask for a referral
☐ Ask a question
☐ Prescription refill
☐ Other ____________________
☐ No interaction with clinic in last 6 months

6. Based on your response to question 5 above, what is your OVERALL rating of your most recent clinic interaction? *(check only one)*

Poor ☐ Fair ☐ Good ☐ Very good ☐ Excellent ☐ No interaction ☐

7. **For which of the following services would you prefer to use a secure website such as MDO rather than the telephone?** *(check all that apply)*

☐ Communicate non-urgent messages for doctors and/or nurse
☐ Refill prescriptions
☐ Request referrals
☐ Schedule appointments
☐ Other ________________

8. Did you ever use My Doctor's Office (MDO)?

Yes ☐ No ☐

If NO, please SKIP Questions 9 through 12 and GO TO Question 13

9. How many times in the past 6 months did you log on to MDO website?

0 ☐ 1 ☐ 2 ☐ 3 ☐ more than 3 ☐ _____ if more than 3 please indicate how many times

10. How satisfied are you with your experience using MDO online services? *(check only one)*

☐	☐	☐	☐	☐
Very dissatisfied	Dissatisfied	Neutral	Satisfied	Very Satisfied

11. Did using MDO online services ever save you a phone call to the clinic.

Yes ☐ No ☐

12. Did using MDO online services ever save you a visit to the clinic?

Yes ☐ No ☐

13. Based on your experiences using the <u>phone</u> or <u>MDO</u> to contact the Garfield clinic, please rate the services below. ***(check only one for each task)***

a) **Communicating non-urgent messages for doctors and/or nurses.**

Poor ☐ Fair ☐ Good ☐ Very good ☐ Excellent ☐ Never did this ☐

b) **Refilling prescriptions.**

Poor ☐ Fair ☐ Good ☐ Very good ☐ Excellent ☐ Never did this ☐

c) **Requesting referrals.**

Poor ☐ Fair ☐ Good ☐ Very good ☐ Excellent ☐ Never did this ☐

d) **Scheduling appointments.**

Poor ☐ Fair ☐ Good ☐ Very good ☐ Excellent ☐ Never did this ☐

14. OVERALL, would you prefer to use MDO or telephone when communicating non-urgent messages with the clinic?

☐	☐
Online services like MDO	Telephone

15. How likely is it that you will use My Doctor's Office online services in the future? *(check only one)*

☐	☐	☐	☐	☐
Very unlikely	Unlikely	Neutral	Likely	Very likely

16. Compared with the beginning of the study, would you say that your communication with the clinic is…

☐	☐	☐	☐	☐
A lot worse	A little worse	Neither better nor worse	A little better	A lot better

17. OVERALL, how would you rate the services you receive from the Garfield Clinic? *(check only one)*

Poor ☐ Fair ☐ Good ☐ Very good ☐ Excellent ☐

18. Currently, there is no cost to you for your electronic correspondence with your doctor. However, as this technology becomes more commonplace, some offices may begin charging for this service. What would you personally be willing to pay for each completed electronic correspondence with your doctor? Please write a dollar amount below.

$____________________

19. We are interested in your feedback about how to improve our services. We would like to know about your experiences using My Doctor's Office online services. Please write in the box below.

20. You have my approval to review the phone and electronic messages in my medical record to determine overall message patterns in this study. No personal information will be maintained by the study researchers.

☐ I consent to researchers reviewing my messages

I do not consent to researchers reviewing my messages

You have successfully completed this questionnaire.

Thank you for your participation.
