# Supplementary material for: An Internet-Based Patient-Provider Communication System: Randomized Controlled Trial
Source: J Med Internet Res. 2005 Aug 5;7(4):e47. doi: 10.2196/jmir.7.4.e47 (PMC1550679; doi:10.2196/jmir.7.4.e47)
Supplement: Supplementary file 4 [file jmir_v7i4e47_app4.pdf]

My Doctor's Office (MDO) Electronic Survey #2.135C   Follow up Survey Controls

First Name [ ]   Last Name [ ]

Email [ ]

The following questions relate to your interaction with the clinic. Point the arrow and click the box that represents the best answer.

1. **In the past 6 months, how many times did you call the clinic?**

   0 □   1 □   2 □   3 □   more than 3 □

2. **In the past 6 months how many times did you send email to the clinic staff or your physician?**

   0 □   1 □   2 □   3 □   more than 3 □

3. **In the past 6 months, how many times were you seen in the clinic by either a doctor or nurse?**

   0 □   □1   2 □   3 □   more than 3 □

4. **In the past 6 months, what was your most recent interaction with the clinic, excluding a doctor visit?** *(check only one)*

   □ Phone call
   □ Electronic-mail (e-mail)
   □ Other ___________________________
   □ Did not contact the clinic in the past 6 months

5. **What was your main reason for contacting the clinic?** *(check only one)*

   □ Schedule an appointment
   □ Ask for a referral
   □ Ask a question
   □ Prescription refill
   □ Other ___________________________
   □ No interaction with clinic in last 6 months

6. **Based on your response to question 5 above, what is your OVERALL rating of your most recent clinic interaction?** *(check only one)*

   Poor □   Fair □   Good □   Very good □   Excellent □   No interaction □

7. **For which of the following services would you prefer to use a secure website such as My Doctor's Office rather than the telephone?** *(check all that apply)*

   □ Communicate non-urgent messages for doctors and/or nurse
   □ Refill prescriptions
   □ Request referrals
   □ Schedule appointments
   □ Other

8. **OVERALL, would you prefer to use MDO or telephone when communicating non-urgent messages to the clinic?** (check only *one)*

☐ Online services Like My Doctor's Office	☐ Telephone

9. Based on your experiences interacting with the Garfield clinic, please rate the services below. *(check only one for each task)*

a) **Communicating non-urgent messages to doctors and/or nurses.**

Poor ☐ Fair ☐ Good ☐ Very good ☐ Excellent ☐ Never did this ☐

b) **Refilling prescriptions.**

Poor ☐ Fair ☐ Good ☐ Very good ☐ Excellent ☐ Never did this ☐

c) **Requesting referrals.**

Poor ☐ Fair ☐ Good ☐ Very good ☐ Excellent ☐ Never did this ☐

d) **Scheduling appointments.**

Poor ☐ Fair ☐ Good ☐ Very good ☐ Excellent ☐ Never did this ☐

10. **OVERALL, how would you rate the services you receive from the Garfield Clinic?** *(check only one)*

Poor ☐ Fair ☐ Good ☐ Very good ☐ Excellent ☐

11. Currently, there is no cost to you for your electronic correspondence with your doctor. However, as this technology becomes more commonplace, some offices may begin charging for this service. What would you be willing to pay for each completed electronic correspondence with your doctor? Please write a dollar amount below.

$____________________

12. Compared with the beginning of the study, would you say that your communication with the clinic is…

A lot worse	A little worse	Neither better nor worse	A little better	A lot better

13. We are interested in your feedback about how to improve our services. We would like to know about your experiences interacting with the Garfield Clinic. Please write in the box below.

14. You have my approval to review the phone messages in my medical record to determine overall message patterns in this study. No personal information will be maintained by the study researchers.

☐ I consent to researchers reviewing my messages	I do not consent to researchers reviewing my messages

You have successfully completed this questionnaire.

Thank you for your participation.
