# Supplementary material for: An Internet-Based Patient-Provider Communication System: Randomized Controlled Trial
Source: J Med Internet Res. 2005 Aug 5;7(4):e47. doi: 10.2196/jmir.7.4.e47 (PMC1550679; doi:10.2196/jmir.7.4.e47)
Supplement: Supplementary file 5 [file jmir_v7i4e47_app5.pdf]

Satisfaction with Last Clinic Interaction: Did Not Complete vs. Completed Study

Question	Did not complete study n=265 n (%)	Completed Study n=341 n (%)
Based on method of and reason for contact with clinic, what is your overall rating of your most recent clinic interaction?		
Missing	27 (10%)	49 (14%)
Poor	28 (11%)	23 (7%)
Fair	59 (22%)	60 (18%)
Good	67 (25%)	72 (21%)
Very Good	52 (20%)	85 (25%)
Excellent	32 (12%)	52 (15%)

Satisfaction with Last Clinic Interaction: Control vs. Portal Group

Question	Control Group n=301 n (%)	Portal Group n=305 n (%)
Based on method of and reason for contact with clinic, what is your overall rating of your most recent clinic interaction?		
Missing	33 (11%)	43 (14%)
Poor	22 (7%)	29 (10%)
Fair	59 (20%)	60 (20%)
Good	72 (24%)	67 (22%)
Very Good	75 (25%)	62 (20%)
Excellent	40 (13%)	44 (14%)

Patient satisfaction ratings at the end of 6-month study period

Question	Portal Group Overall n=175 n (%)	Portal Group Non-user n=98 n (%)	Control n=166 n (%)
Compared with the beginning of the study, would you say that your communication with the clinic is…			
A lot worse	2 (1%)	2 (2%)	5 (3%)
A little worse	2 (1%)	1 (1%)	8 (5%)
Neither worse nor better	93 (53%)	66 (67%)	115 (79%)
A little better	39 (22%)	16 (16%)	13 (9%)
A lot better	38 (22%)	13 (13%)	5 (3%)
Overall, how would you rate the services you receive from the clinic?			

Question	Portal Group Overall n=175 n (%)	Portal Group Non-user n=98 n (%)	Control n=166 n (%)
Poor	4 (2%)	2 (2%)	7 (4%)
Fair	14 (8%)	11 (11%)	25 (15%)
Good	53 (30%)	36 (37%)	52 (32%)
Very Good	55 (32%)	28 (29%)	51 (31%)
Excellent	48 (28%)	21 (21%)	27 (17%)
Based on your experiences using the phone or the portal to contact the clinic, please rate the services below:			
Communicating non-urgent messages to doctor and/or nurse	n=141	n=76	n=137
Poor	11 (8%)	9 (12%)	18 (13%)
Fair	26 (18%)	19 (25%)	39 (28%)
Good	27 (19%)	16 (21%)	37 (27%)
Very Good	36 (26%)	16 (21%)	31 (23%)
Excellent	41 (29%)	16 (21%)	12 (9%)
Refilling prescription	n=95	n=45	n=118
Poor	6 (6%)	2 (4%)	10 (8%)
Fair	12 (13%)	7 (16%)	23 (19%)
Good	17 (18%)	12 (27%)	33 (28%)
Very Good	25 (26%)	15 (33%)	33 (28%)
Excellent	35 (37%)	9 (20%)	19 (16%)
Requesting referrals	n=80	n=43	n=106
Poor	5 (6%)	1 (2%)	12 (11%)
Fair	12 (15%)	8 (19%)	17 (16%)
Good	13 (16%)	10 (23%)	33 (31%)
Very Good	22 (28%)	7 (16%)	24 (23%)
Excellent	28 (35%)	17 (40%)	20 (19%)
Scheduling appointments	n=131	n=70	n=154
Poor	13 (10%)	8 (11%)	13 (8%)
Fair	24 (18%)	17 (24%)	34 (22%)
Good	23 (18%)	14 (20%)	60 (39%)
Very Good	31 (24%)	16 (23%)	25 (16%)
Excellent	40 (31%)	15 (21%)	22 (14%)

Portal Users Satisfaction

Question	Portal Users<br>n=114<br>n (%)
If answered Yes to having used MDO, how satisfied are you with MDO online services?	
Very dissatisfied	4 (4%)
Dissatisfied	9 (8%)
Neutral	20 (18%)
Satisfied	37 (33%)
Very satisfied	43 (38%)

Portal Users Satisfaction

Question	Entire Portal Group<br>n=175<br>n (%)
How likely is it that you will use MDO services in the future?	
Very unlikely	9 (5%)
Unlikely	13 (7%)
Neutral	20 (11%)
Likely	52 (30%)
Very likely	80 (46%)
